# Use of explainable cluster analysis to identify distinct subtypes of Meige syndrome patients and associated biomarker profiles

**DOI:** 10.3389/fimmu.2026.1904682

**Published:** 2026-07-30

**Authors:** Xiaoli Sun, Xinyu Feng, Yingjie Zhu, Runing Fu

**Affiliations:** 1 Department of Laboratory Medicine, The First Affiliated Hospital of Henan University of Chinese Medicine, Zhengzhou, China; 2 Department of Laboratory Medicine, The Third People’s Hospital of Henan Province, Zhengzhou, China

**Keywords:** biomarkers, explainable clustering analysis, machine learning, Meige syndrome, SHAP

## Abstract

**Background:**

Meige syndrome (MS), a complex form of segmental craniocervical dystonia, currently lacks objective diagnostic biomarkers.This study aims to identify biological biomarkers that can stratify patients with MS and to reveal the complex pathophysiological heterogeneity of this disorder, with particular attention to the peripheral immune-metabolic profiles that may reflect underlying neuroimmune dysfunction.

**Methods:**

We retrospectively collected clinical and laboratory data from 1,782 patients with MS during their first hospitalization at the MS Center of The Third People’s Hospital of Henan Province between 2023 and 2025. A total of 66 clinical and laboratory indicators were included. An interpretable clustering analysis framework was applied to perform a series of pipeline analyses, including imputation, dimensionality reduction, outlier removal, and clustering. Next, feature importance was assessed. A multi-class classification model was then constructed and evaluated using XGBoost. Finally, the Shapley Additive exPlanations(SHAP) algorithm was employed to interpret the contribution of each feature.

**Results:**

The preprocessing pipeline effectively imputed missing values (ranging from 0.06% to 14.14%) and removed outliers. Unsupervised clustering analysis revealed three heterogeneous subtypes of MS. A total of 23 important features were selected to construct a multi-class XGBoost classification model. The model demonstrated excellent performance, with a macro-average AUC of 0.9790 and an Obuchowski index of 0.9784. The SHAP analysis further elucidated the unique hematological and biochemical metabolic characteristics of each subtype.

**Conclusion:**

This study establishes a novel, data-driven subtyping system for MS based on objective blood and metabolic profiles. The three identified subtypes display distinct immune-metabolic signatures: a hypercoagulable-inflammatory profile, a metabolic-excess profile with impaired vascular protection, and a frailty-associated antioxidant-deficient profile. These peripheral biomarker patterns suggest testable hypotheses regarding neuroimmune mechanisms—including fibrinogen-driven microglial activation, oxidized lipid-mediated blood-brain barrier disruption, and uric acid-dependent antioxidant depletion—that may contribute to the pathophysiology of different MS subtypes. This framework may inform future prospective investigations into precision medicine approaches for MS, though substantial validation is required before clinical translation.

## 1 Introduction

Meige syndrome (MS), also known as idiopathic cranio-cervical dystonia, is a debilitating segmental dystonia characterized by involuntary contractions of eyelid muscles (blepharospasm) and lower facial/oromandibular muscles (oromandibular dystonia) (1–3). The condition predominantly affects middle-aged and elderly individuals, with a female predominance (female-to-male ratio approximately 2~3:1) (4). MS significantly impairs patients’ quality of life, affecting visual function, speech articulation, social interactions, and psychological well-being (5–7). Despite the characteristic clinical manifestations, the etiology and pathogenesis of MS remain incompletely understood, with current hypotheses implicating basal ganglia dysfunction, neurotransmitter imbalance, genetic predisposition, and environmental factors (8, 9).

Current clinical diagnosis and classification of MS primarily rely on phenomenological observation of affected muscle groups, categorizing patients into blepharospasm-dominant, oromandibular dystonia-dominant, and mixed subtypes (10).

Currently, there is no curative treatment for MS. The primary goal is to control symptoms and improve quality of life. Treatment options include antipsychotic drugs and agents that modulate the dopaminergic and cholinergic systems. Surgical interventions, such as facial nerve avulsion and resection of periorbital muscles, are also used. Local injection of botulinum toxin type A (BTX-A) is another common approach. In addition, advanced therapeutic technologies have emerged in recent years, including deep brain stimulation (DBS), and neural circuit blocking (NCB).

However, the effectiveness of these treatments varies considerably among patients. Currently, precise treatment is not achievable because the pathogenesis of MS remains incompletely understood. The existing classification of MS relies solely on clinical symptoms and does not account for potential heterogeneity at the pathological and physiological levels. Meanwhile, accumulating evidence indicates that neurological disorders such as Parkinson’s disease and amyotrophic lateral sclerosis exhibit substantial molecular and pathological heterogeneity. In these diseases, symptom-based classification alone may obscure critical biological subtypes (11, 12).

Accumulating evidence implicates neuroimmune interactions in the pathogenesis of dystonia and related movement disorders. Peripheral inflammatory biomarkers—including the neutrophil-to-lymphocyte ratio (NLR), platelet-to-lymphocyte ratio (PLR), and systemic immune-inflammation index (SII)—have been associated with disease severity and progression in Parkinson’s disease (13). Furthermore, recent studies demonstrate that circulating coagulation factors, particularly fibrinogen, can directly activate microglia upon extravasation across a compromised blood-brain barrier (BBB), establishing a direct molecular link between peripheral blood profiles and central neuroinflammation (14, 15). Oxidized lipids, uremic toxins, and uric acid have each been shown to modulate BBB integrity and neuroimmune signaling through distinct pathways (16–18). These observations suggest that the routine blood biomarkers available in clinical practice may carry information about the neuroimmune state of individual patients—information that has not been systematically leveraged in the classification of MS.

Thus, in this study, we integrated a comprehensive set of demographic, clinical, and routine laboratory parameters from a large cohort of MS patients to perform an unsupervised clustering analysis (19). The main objective was to explore the data-driven, biologically homogeneous subtypes that may reflect distinct underlying pathophysiological mechanisms. We then interpreted the clustering results using *post-hoc* feature importance analysis with an interpretable algorithm (e.g., XGBoost). This allowed us to pinpoint the key biomarkers defining each subtype. This approach successfully revealed three robust phenotypic clusters. These findings are significant because they transform complex, high-dimensional clinical data into actionable biological insights—specifically, the identification of three distinct pathological patterns. Our work provides a foundation for future prospective studies on precision medicine in MS.

## 2 Materials and methods

### 2.1 Ethics statement

This study was conducted in accordance with the Declaration of Helsinki. It was approved by the Ethics Committee of The Third People’s Hospital of Henan Province (approval number: 2025SZSYLCYJ1208). As a retrospective study, the requirement for informed consent was waived.

### 2.2 Study population and data collection

This study included patients who were first diagnosed with MS and hospitalized at the MS Center of The Third People’s Hospital of Henan Province from July 31, 2023 to December 31, 2025. Inclusion criteria were a clinical diagnosis of Meige syndrome in newly treated inpatients. Exclusion criteria included acute or chronic infections, severe comorbidities (e.g., significant cardiac, liver, kidney, or immune system dysfunction), and substantial missing data on characteristic variables.

A total of 1,782 patients were initially enrolled, and 66 clinical indicators were extracted. These included demographic characteristics and laboratory indicators.

### 2.3 Data preprocessing

To ensure the validity and reliability of the data analysis, we employed a preprocessing framework (19). The missing data imputation was performed in a strict sequential four-step pipeline:

1. High-missingness variable removal: Variables with a missing rate exceeding a predefined threshold were removed.
2. Pairwise model-based imputation: For each variable pair with strong correlation, imputation was conducted by three cases: (a) numerical–numerical via linear regression; (b) categorical–categorical via hot-deck random sampling; (c) mixed type by discretizing the numerical variable and treating as categorical.
3. High-missingness observation removal: Observations with a missing proportion exceeding the default 1/3 were deleted.
4. Within-cluster KNN hot-deck imputation: Variable clusters were first identified using mutual information and graph-theoretic connected components. Within each cluster, a K-nearest neighbor (k = 8) hot-deck algorithm was applied to search for donors, using the Euclidean distance with missing values (20, 21).

This framework also included outlier detection, feature dimensionality reduction, and unsupervised clustering. The Isolation Forest method was used to detect outliers in the high-dimensional data. This step was performed to prevent distortion of the clustering and classification results. As a result, 1,756 patients were retained. Finally, integrated dimensionality reduction and clustering analysis were conducted on both numerical and categorical variables (22).

Three mainstream clustering algorithms were compared: K-Means, Agglomerative Clustering, and Gaussian Mixture Models. The integration of multiple clustering algorithms through consensus approaches enhances the robustness and reliability of the number of clusters. The Within-Cluster Sum of Squares (WSS), a standardized weighted squared distance was employed to evaluate the optimal number of clusters. The number of possible clusters (k) was to range from 2 to 9 to avoid an excessive numbers of clusters that would not be clinically useful. Additionally, To evaluate cluster stability, we performed 30 bootstrap resampling iterations.

### 2.4 Feature selection

To construct a parsimonious and predictive model, a four-step feature selection procedure was implemented. Firstly, features with statistical significance were selected based on univariate analysis (significance threshold set at p<0.05). Subsequently, Cohen’s d for numerical variables or Cramér’s V for categorical variables was calculated based on the comparison between the two groups, and only those with |d|≥0.5 (indicating a large effect size) were retained. Then, Pearson correlation coefficients were used to assess collinearity between predictors, those with a Pearson correlation coefficient greater than 0.78 were excluded. Finally, a random forest classifier was used in combination with Recursive Feature Elimination (RFE) and 5-fold cross-validation over 100 iterations. This process eliminated unimportant features and retained the optimal feature subset.

### 2.5 Model construction and evaluation

To identify patterns strongly associated with the cluster, we employed XGBoost, a powerful and efficient gradient boosting algorithm, to perform multi-class classification. The dataset was split into training and test sets using stratified sampling at an 8:2 ratio. Hyperparameter optimization was achieved via grid search combined with 5-fold cross-validation. The optimal parameter combination was determined as follows: learning_rate = 0.1, max_depth = 3, n_estimators = 300.

On the test set, the multi-class classification performance was evaluated using accuracy, precision, recall, F1-score, the macro-average ROC curve, the Obuchowski index, and a confusion matrix. The macro-average ROC curve was obtained by averaging the binary ROC curves for each class against the remaining classes. The Obuchowski index is a diagnostic accuracy metric designed for multi-class problems (23, 24). It is calculated based on pairwise comparisons among classes. The index ranges from 0 to 1, with higher values indicating better overall diagnostic performance. It is also robust to class imbalance.

### 2.6 SHapley Additive exPlanations(SHAP)

We applied the SHAP algorithm to explain the black-box nature of the XGBoost model and to identify biologically meaningful biomarkers. For global interpretability, the mean absolute SHAP value was calculated for each class to determine the importance ranking of features. For local interpretability, a beeswarm plot and a heatmap were used to visualize the SHAP values. These visualizations illustrated how specific feature values influenced the predicted probabilities of MS subtypes (MS1, MS2 and MS3) at the individual sample level.

### 2.7 Webpage deployment tool based on streamlit framework

To facilitate the utility of the model in clinical settings, optimized predictive model was implemented into a web application established based on the Streamlit Python-based framework. When the values of corresponding features from the XGBoost model are provided, the application can return the probability of MS subtypes (MS1, MS2 and MS3) and a waterfall plot for the individual.

### 2.8 Statistical analysis

Data analyses were conducted using R version 4.3.2 (https://www.r-project.org) and Python version V.3.9.18 (https://www.python.org). The normality of the data was assessed using the Shapiro–Wilk test. A Student’s t-test or Welch’s t-test employed to determine any differences between the two sets. Continuous variables with skewed distributions were presented as median with interquartile range and compared using the Mann-Whitney U test or Kruskal-Wallis H test. Categorical variables were presented as numbers with percentages and compared using the Chi-square test or Fisher’s exact test. A two-tailed P value <0.05 was considered statistically significant. P value less than 0.05 after multiple comparisons correction using Benjamini-Hochberg false discovery rate (FDR) method was considered significant.

The ML algorithms were implemented with libraries of XGBoost (V.2.1.4) and scikit-learn (V.1.6.1) in Python. Model visualisations were performed using Shap library (V.0.49.1). In addition, we established a web page application tool based on the Streamlit package (Version 1.50.0).

## 3 Results

### 3.1 An overview of the study can be found in **Figure 1**

**Figure 1:**
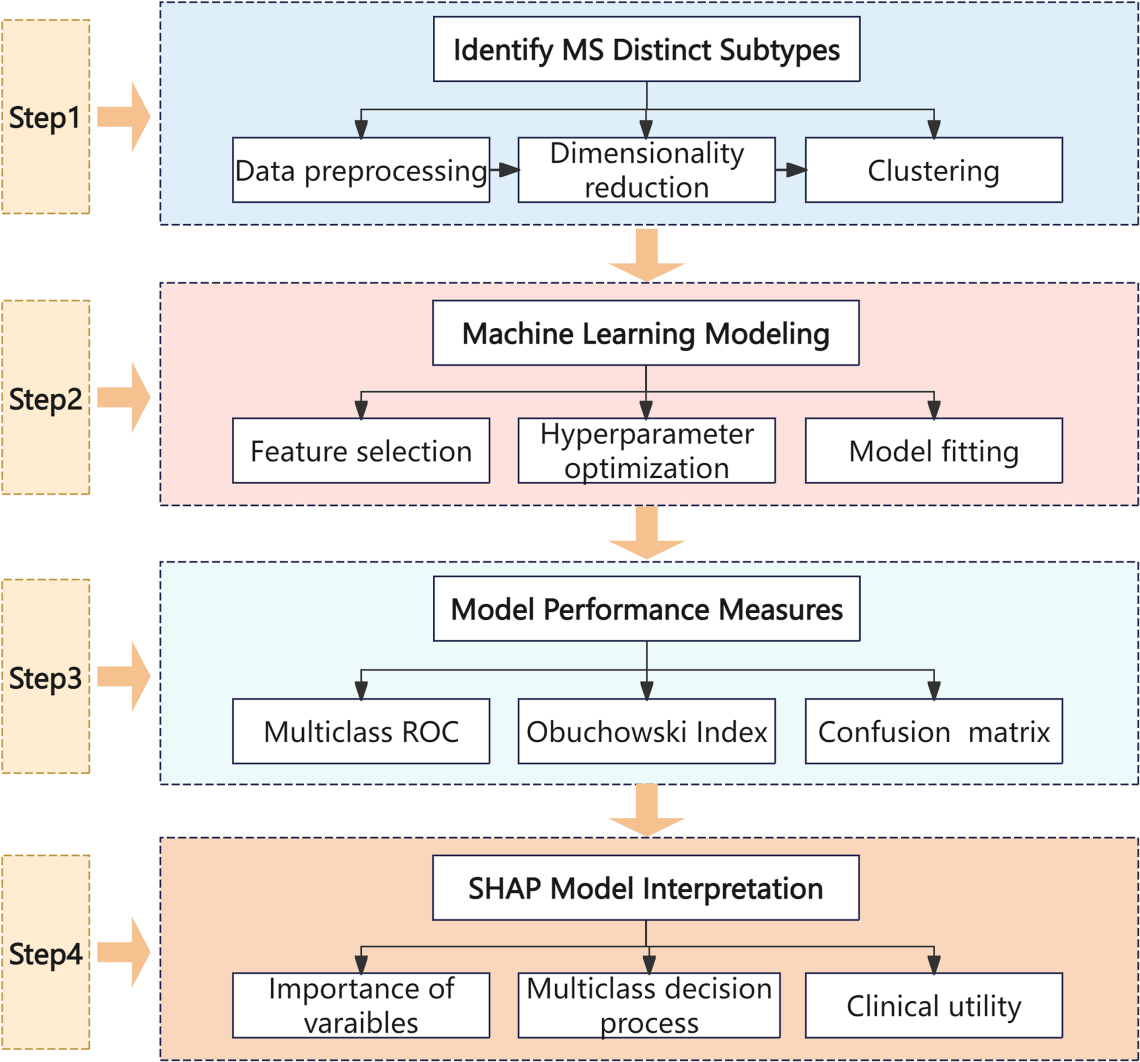
Flowchart of the study process.

### 3.2 Patient characteristics and clustering results

**Figure 2:**
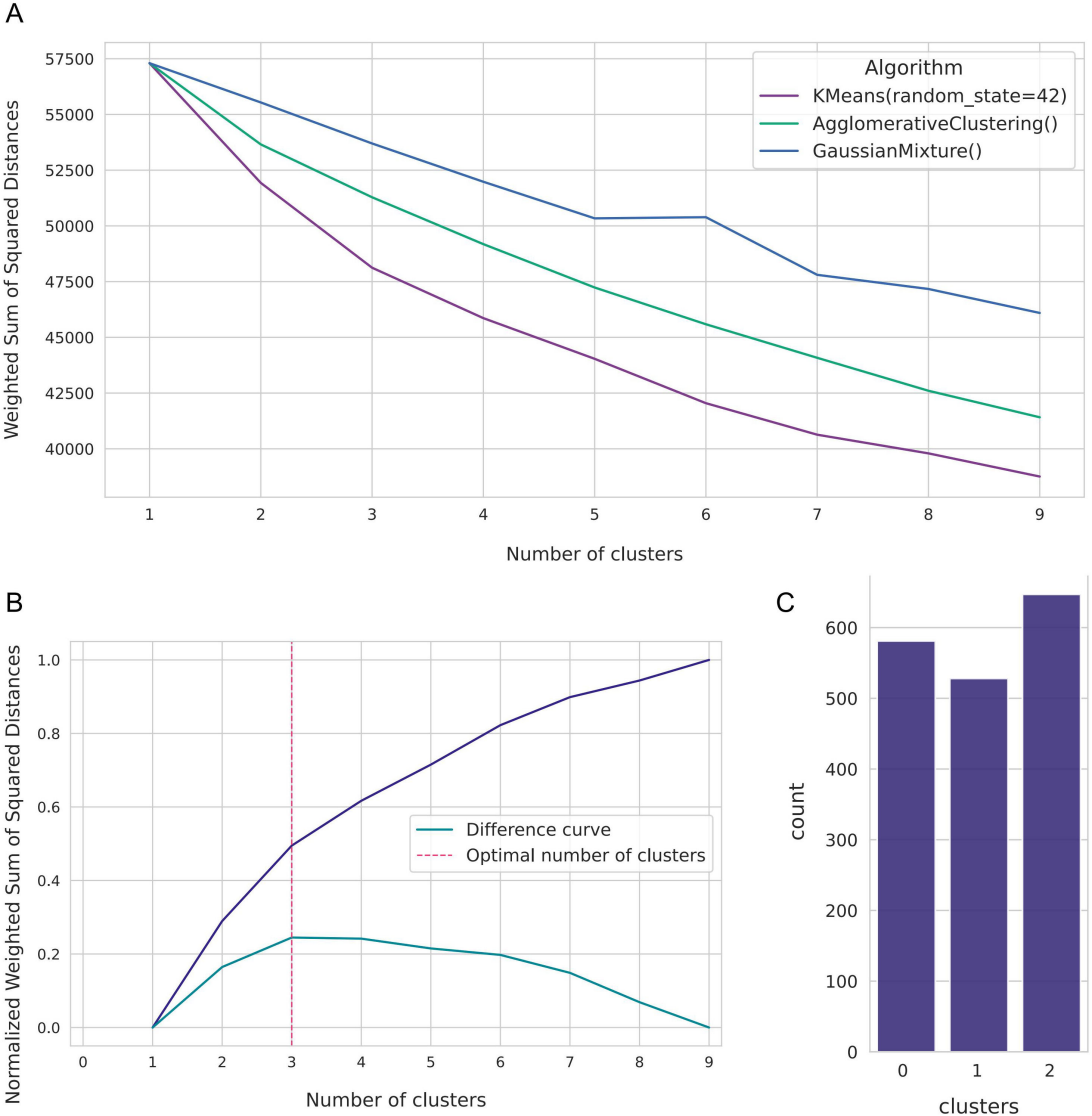
Clustering algorithm determines the optimal number of clusters.

After rigorous data preprocessing, 1,756 patients were included in the final study. A total of 66 features were analyzed, of which 60 contained missing values. The missing rates ranged from 0.06% to 14.14% (**Supplementary Table 1**; **Supplementary Figure 1**). No significant differences were observed in feature distributions before and after imputation (**Supplementary Figure 2**). To identify potential subtypes of MS, an interpretable clustering algorithm successfully divided the cohort into three distinct subgroups: MS1, MS2, and MS3 (**Figure 2**). The bootstrap-based stability analysis also demonstrates that k = 3 is optimal, balancing cluster quality, stability, and interpretability (**Supplementary Figure 3**).Significant differences in demographic and clinical baseline characteristics were found among the three sugroups (**Table 1**). These results indicate heterogeneity within the MS population that cannot be captured by existing classification systems based on anatomical characteristics of involved muscle groups and symptom combination patterns.

**Table 1:** Baseline characteristics according to clusters of Meige syndrome patients.

Characteristics	MS1 (n=581)	MS2 (n=528)	MS3 (n=647)	pvalue
Age	59 (54, 65)	60 (54, 67)	60 (53, 66)	0.683
Sex, n (%)				< 0.001
Male	102 (17.6%)	402 (76.1%)	40 (6.2%)	
Female	479 (82.4%)	126 (23.9%)	607 (93.8%)	
BMI kg/m2	25.3 (23.2, 27.5)	25.45 (23.7, 27.5)	23.1 (21.25, 25.3)	< 0.001
SBP mmHg	129 (121, 137)	127 (119, 135)	122 (114, 130)	< 0.001
DBP mmHg	81 (76, 85)	81 (76.75, 86)	76 (72, 81)	< 0.001
Pulse	74 (73, 77)	74 (72, 76)	74 (72, 76)	< 0.001
Hospital stay	3 (2, 6)	3 (2, 5)	3 (2, 6)	0.051
Surgical history n (%)				0.105
Yes	348 (19.8%)	319 (18.2%)	422 (24%)	
No	233 (13.3%)	209 (11.9%)	225 (12.8%)	
WBC ×10^9/L	5.45 (4.72, 6.5)	5.55 (4.7, 6.31)	4.67 (3.96, 5.48)	< 0.001
RBC ×10^12/L	4.34 (4.03, 4.66)	4.49 (4.1675, 4.8)	4.05 (3.81, 4.35)	< 0.001
PLT ×10^9/L	231 (203, 265)	186 (157, 221)	199 (170.5, 232)	< 0.001
HGB g/L	129 (123, 138)	141 (133, 149)	121 (115, 127)	< 0.001
HCT %	39.2 (37.2, 41.7)	42.05 (39.7, 44.4)	36.7 (34.85, 38.7)	< 0.001
MCV fL	90.4 (88.3, 93.1)	92.3 (90, 94.5)	90.6 (88.1, 93.25)	< 0.001
MCHC g/L	331 (326, 335)	335 (330, 339)	328 (323, 333)	< 0.001
MCH pg	30 (29, 30.9)	30.9 (30, 31.7)	29.8 (28.7, 30.8)	< 0.001
Neutrophil ×10^9/L	3.08 (2.51, 3.83)	3.2 (2.68, 3.83)	2.58 (2.04, 3.15)	< 0.001
Lymphocyte ×10^9/L	1.91 (1.6, 2.29)	1.76 (1.46, 2.15)	1.63 (1.365, 1.92)	< 0.001
Monocyte ×10^9/L	0.26 (0.21, 0.32)	0.3 (0.24, 0.35)	0.23 (0.19, 0.27)	< 0.001
Basophils ×10^9/L	0.03 (0.02, 0.03)	0.02 (0.02, 0.03)	0.02 (0.02, 0.03)	< 0.001
Eosinophils ×10^9/L	0.11 (0.07, 0.16)	0.14 (0.09, 0.21)	0.1 (0.06, 0.15)	< 0.001
RDW-CV	12.7 (12.3, 13.1)	12.8 (12.4, 13.1)	12.7 (12.3, 13.2)	0.094
RDW-SD	41.2 (39.9, 42.8)	42.2 (40.8, 43.6)	41.5 (40.1, 42.8)	< 0.001
PDW	16 (15.8, 16.3)	16.2 (15.9, 16.4)	16.1 (15.8, 16.3)	< 0.001
PCT %	0.23 (0.2, 0.26)	0.19 (0.16, 0.216)	0.20 (0.18, 0.23)	< 0.001
P-LCR %	23.4 (19.5, 30.2)	25.8 (20.8, 32.2)	26.2 (21, 31.9)	< 0.001
MPV fL	9.7 (9.1, 10.6)	10 (9.3, 11)	10.1 (9.3, 10.9)	< 0.001
LDL-C mmol/L	3.25 (2.87, 3.65)	2.44 (1.97, 2.91)	2.36 (1.99, 2.80)	< 0.001
TC mmol/L	5.46 (5.01, 6.04)	4.17 (3.61, 4.80)	4.37 (3.86, 4.92)	< 0.001
TG mmol/L	1.82 (1.33, 2.47)	1.51 (1.1, 2.06)	1.17 (0.92, 1.54)	< 0.001
HDL-C mmol/L	1.31 (1.13, 1.5)	1.1 (0.94, 1.26)	1.38 (1.18, 1.61)	< 0.001
sdLDL-C mmol/L	0.96 (0.68, 1.21)	0.675 (0.46, 0.94)	0.48 (0.36, 0.67)	< 0.001
ApoA1	1.22 (1.08, 1.36)	1 (0.88, 1.14)	1.17 (1.04, 1.33)	< 0.001
ApoB	0.98 (0.88, 1.11)	0.75 (0.64, 0.87)	0.73 (0.63, 0.83)	< 0.001
ALT U/L	19 (15, 28)	20 (15, 29)	15 (12, 21)	< 0.001
AST U/L	19 (16, 24)	19 (15, 22)	18 (15, 21)	< 0.001
ALP U/L	82 (69, 96)	71 (59, 86)	70 (58.5, 85)	< 0.001
GGT U/L	21 (15, 32)	21 (16, 33)	13 (10, 18)	< 0.001
TP g/L	68.9 (65.7, 72)	65 (62.075, 67.8)	64.7 (61.7, 67.55)	< 0.001
ALB g/L	41.5 (39.8, 43.4)	39.8 (38.2, 41.8)	39.2 (37.5, 41.2)	< 0.001
HbA1c %	5.8 (5.5, 6.1)	5.6 (5.3, 6.1)	5.6 (5.3, 5.9)	< 0.001
FBG mmol/L	5.15 (4.78, 5.69)	5.08 (4.67, 5.7125)	4.76 (4.47, 5.16)	< 0.001
TBIL μmol/L	11.2 (8.5, 14.3)	12.55 (9.8, 16.1)	9.8 (7.4, 13.05)	< 0.001
DBIL μmol/L	2.9 (2.2, 3.8)	3.7 (2.8, 4.8)	2.9 (2.25, 3.9)	< 0.001
TBA μmol/L	4.4 (2.92, 6.81)	4.8 (3.1, 7.3)	4.27 (2.6, 6.73)	0.003
BUN mmol/L	5.28 (4.46, 6.32)	5.74 (4.86, 6.75)	5.18 (4.33, 6.15)	< 0.001
UA μmol/L	296.1 (252.1, 348.3)	335.4 (281.6, 390.9)	248.8 (212.4, 290.8)	< 0.001
Cr μmol/L	53.1 (45.5, 61.4)	67.1 (56.98, 77.23)	52.2 (45.85, 59.6)	< 0.001
K mmol/L	3.96 (3.75, 4.14)	3.9 (3.69, 4.05)	3.91 (3.73, 4.1)	< 0.001
Na mmol/L	142 (140, 143)	142 (141, 143)	142 (141, 143)	0.054
Cl mmol/L	105.2 (103.1, 107)	106 (103.9, 108)	106.9 (104.7, 108.5)	< 0.001
Ca mmol/L	2.26 (2.19, 2.32)	2.19 (2.13, 2.25)	2.19 (2.13, 2.25)	< 0.001
TSH mIU/L	2.16 (1.42, 3.24)	1.803 (1.20, 2.69)	2.058 (1.39, 3.08)	< 0.001
FT3 pmol/L	4.67 (4.33, 5.1)	4.75 (4.40, 5.17)	4.53 (4.19, 4.93)	< 0.001
FT4 pmol/L	10.74 (9.61, 12.59)	11.145 (9.95, 13.01)	10.95 (9.64, 12.87)	0.007
TgAb IU/mL	0.25 (0.25, 1.7)	0.25 (0.25, 1)	0.25 (0.25, 1.77)	0.045
TPOAb IU/mL	1 (0.4, 2)	0.6 (0.3, 1.4)	1 (0.4, 2.55)	< 0.001
PT	10.2 (9.8, 10.6)	10.8 (10.4, 11.3)	10.6 (10.3, 11)	< 0.001
TT	14.4 (13.8, 15)	14.7 (14, 15.3)	15 (14.4, 15.6)	< 0.001
INR	0.92 (0.88, 0.95)	0.97 (0.93, 1.02)	0.95 (0.93, 0.99)	< 0.001
APTT	32 (30.1, 34.5)	32.8 (30.575, 35.6)	32.7 (30.5, 34.85)	< 0.001
FIB g/L	3.06 (2.74, 3.37)	2.73 (2.39, 3.0675)	2.77 (2.46, 3.09)	< 0.001
PS %	87.1 (78.3, 95.4)	84.6 (74.275, 93.6)	75.8 (69.2, 85.8)	< 0.001
PC %	113 (102, 125)	93 (83, 104)	96 (86, 105)	< 0.001
AT-III %	93 (86, 101)	85 (79, 91)	88 (82, 94)	< 0.001
LANR	1.06 (1.01, 1.11)	1.08 (1.02, 1.15)	1.01 (0.96, 1.05)	< 0.001

BMI, Body mass index; SBP, Systolic blood pressure; DBP, Diastolic blood pressure; WBC, White Blood Cell Count; RBC, Red blood cell count; PLT, Platelet Count; HGB, Hemoglobin; HCT, Hematocrit; MCV, Mean Corpuscular Volume; MCHC, Mean Corpuscular Hemoglobin Concentration; MCH, Mean Corpuscular Hemoglobin; NEU, Neutrophil; LYM, Lymphocyte; MON, Monocyte; EOS, Eosinophils; BAS, Basophils; RDW-CV, Red Cell Distribution Width Coefficient of Variation; RDW-SD, Red cell distribution width Standard Deviation; PDW, Platelet Distribution Width; PCT, Plateletcrit; P-LCR, Platelet Larger Cell Ratio; MPV, Mean Platelet Volume; LDL-C, Low-density lipoprotein-cholesterol; TC, Total cholesterol; TG, Triglycerides; HDL-C, High-density lipoprotein-cholesterol; sdLDL-C, Small-dense low-density lipoprotein cholesterol; ApoA1, Apolipoprotein A1; ApoB, Apolipoprotein B; ALT, Alanine aminotransferase; AST, Aspartate aminotransferase; ALP, Alkaline phosphatase; GGT, Gamma glutamyltransferase; TP, Total protein; ALB, Albumin; FBG, Fasting blood glucose; HbA1c, Glycated haemoglobin; TBIL, Total bilirubin; DBIL, Direct bilirubin;TBA, Serum total bile acid; BUN, Blood urea nitrogen; UA, Uric Acid; Cr, Serum creatinine; K, Serum potassium; Na, Serum sodium; Cl, Serum chloride; Ca, Serum calcium; TSH, Thyroid-stimulating hormone; FT3, Free triiodothyronine; FT4, Free thyroxine; TgAb, Anti-thyroglobulin antibodies; TPOAb, Thyroid peroxidase antibodies; LANR, Lupus anticoagulant normalization rate; PT, Prothrombin time; TT, Thrombin time; INR, International normalized ratio; APTT, Activated partial thromboplastin time; FIB, Fibrinogen; PS, Protein S; PC, Protein C; AT-III, Antithrombin III;.

### 3.3 Predictors selection

**Figure 3:**
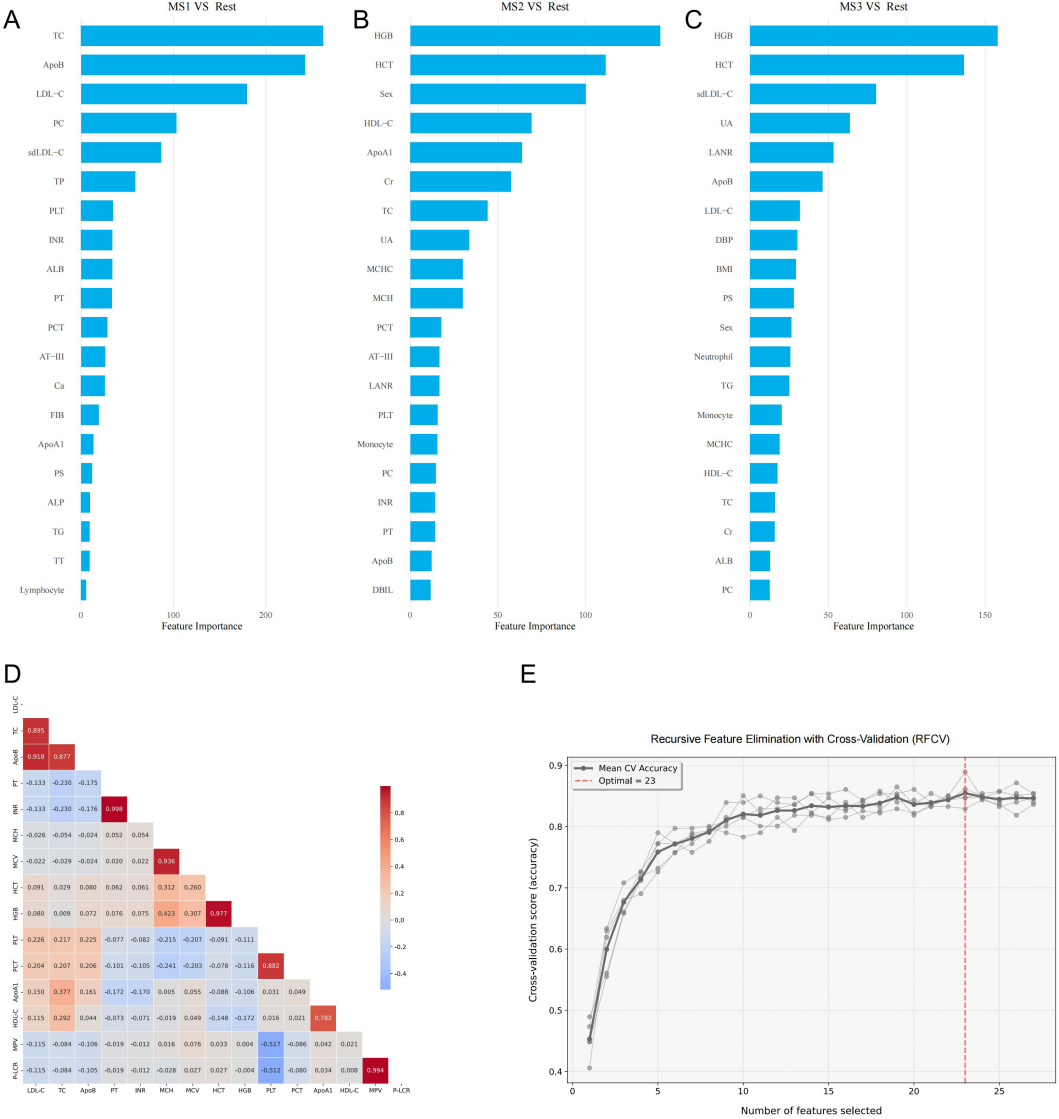
Feature importance analysis. **(A)** MS1 VS Rest **(B)** MS2 VS Rest **(C)** MS3 VS Rest **(D)** Pearson correlation coefficient matrix between variables. **(E)** The recursive elimination algorithm gradually selects the optimal subset of features.

To identify key features, all variable selection was performed on the entire dataset. From an initial pool of 66 candidate variables, we applied a sequential feature selection strategy based on predictive importance. First, univariate analyses identified numerical or categorical features with highly significant differences (all FDR-adjusted p<0.001) between each MS subtype and the rest, many of which also exhibited large effect sizes(e.g., |Cohen’s d|>0.9 for TC, ApoB, LDL-C in MS1; HGB, HCT in MS2/MS3). Then, to reduce redundancy, one variable was removed from each highly collinear pair (Pearson’s r≥0.78), such as HGB-HCT and TC-LDL-C. Finally, Recursive Feature Elimination (RFE) coupled with a random forest classifier and 5-fold cross-validation was applied to the remaining features, yielding a parsimonious and robust feature subset for model construction (**Figure 3**; **Supplementary Tables 2**-**S4**).

**Figure 4:**
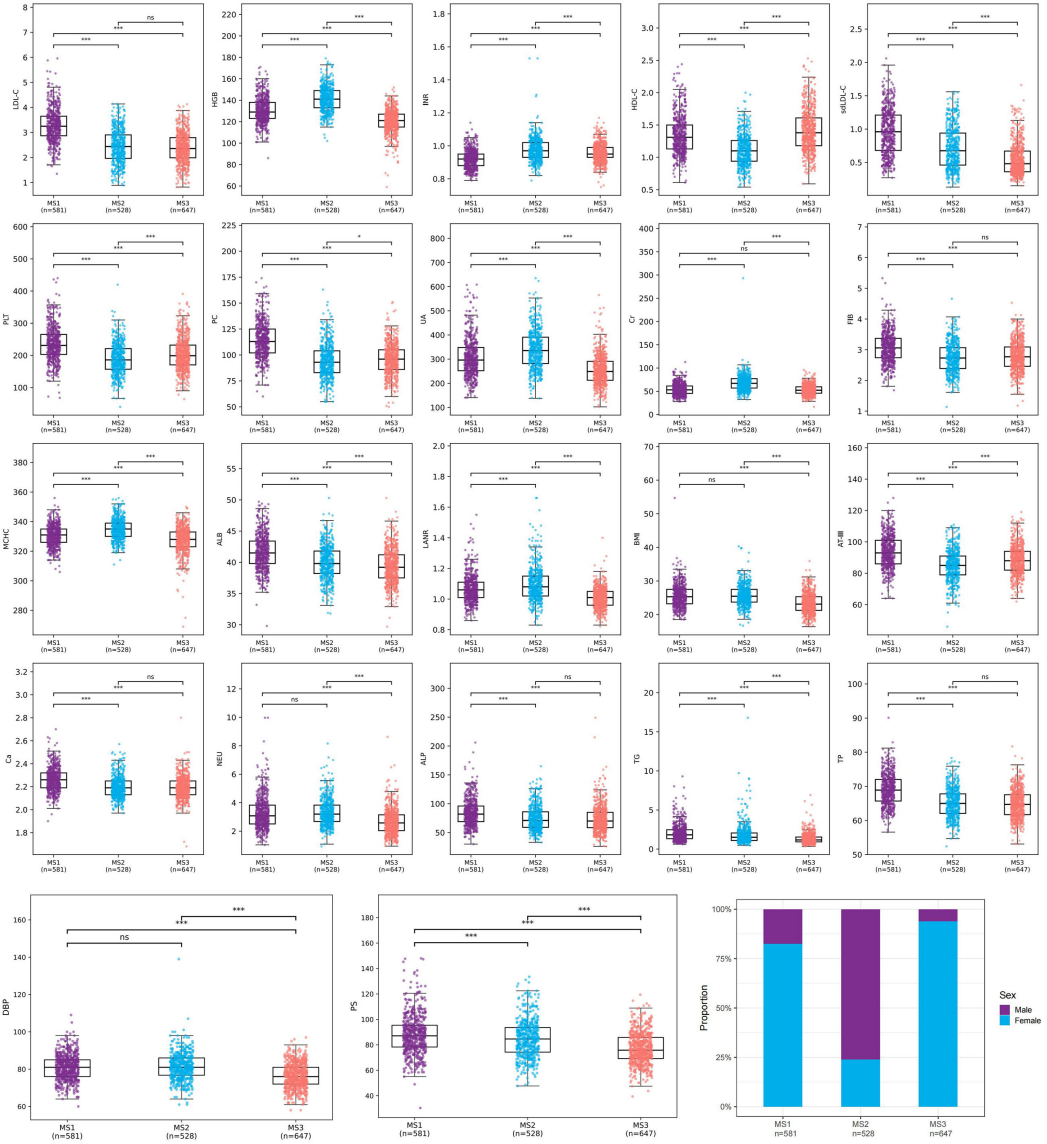
Visualizing 23 key feature distributions across three groups. Statistical significance: *p < 0.05, **p < 0.01, ***p < 0.001, ns, not significant (p > 0.05).

The four-stage feature selection process refined the initial 66 variables to a set of 23 key predictors. These selected features spanned several biological domains (**Supplementary Table 5**): lipid metabolism (LDL-C, HDL-C, sdLDL-C, TG), coagulation (INR, FIB, AT-III, PS, PLT, PC, LANR), renal/metabolic function (UA, Cr, ALB, TP, Ca), hematology (HGB, MCHC, NEU), and demographics (Sex, BMI, DBP). The distributional differences of these features across the three subtypes are detailed in **Figure 4**.

### 3.4 Model development and performance evaluation

**Figure 5:**
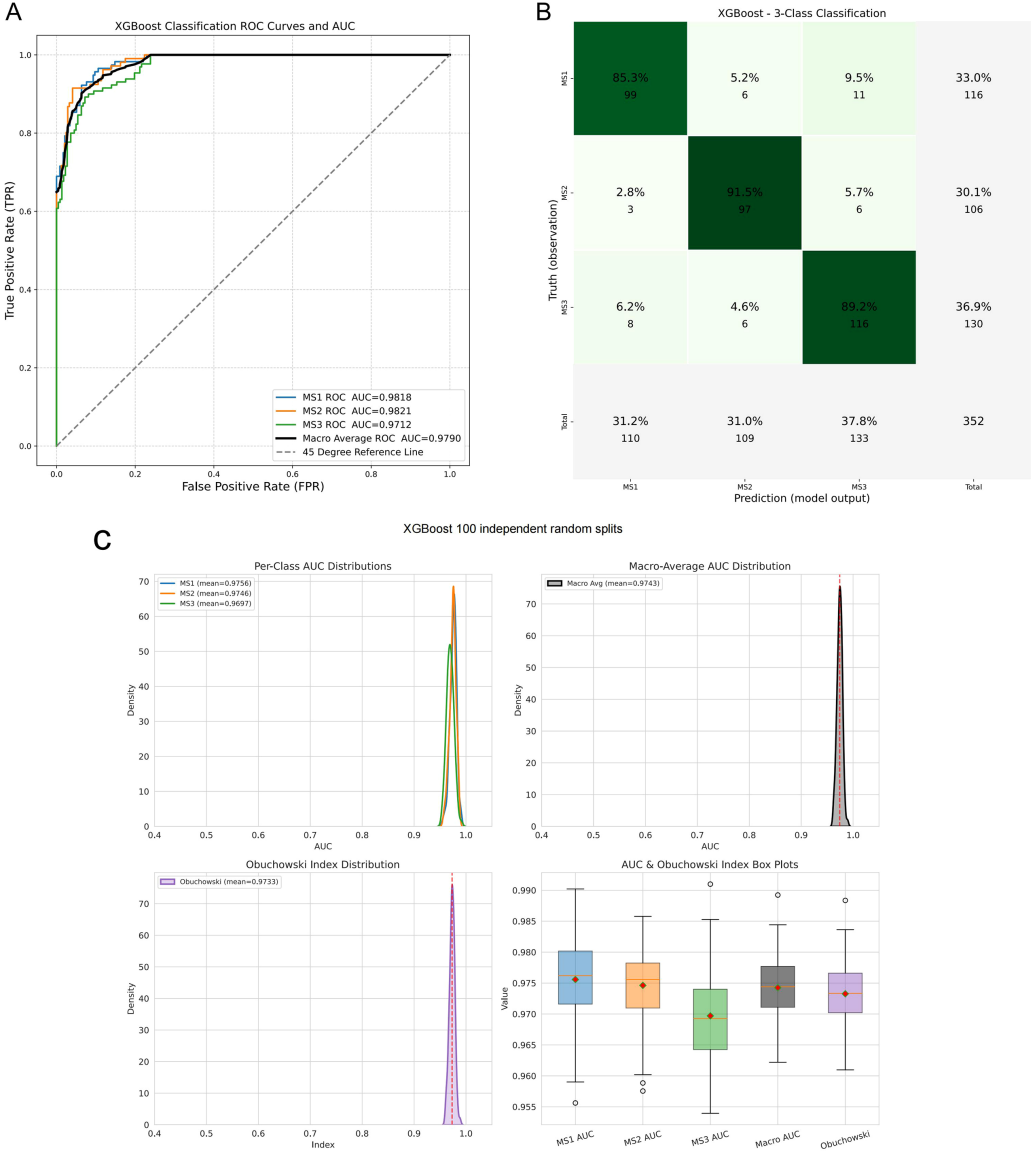
Performance evaluation of multi-class XGBoost model. **(A)** XGBoost Classification. ROC Curves and AUC. **(B)** Confusion matrix heat map. **(C)** AUC curves of XGBoost model with 100 random splits and distribution curves of the Obuchowski index.

An XGBoost model was constructed based on the selected 23 key features to predict the three MS subtypes. On the independent test set, the model achieved an overall accuracy of 89%. Its strong multi-class discrimination capability was evidenced by a macro-average ROC-AUC of 0.9790 (**Figure 5A**). and an Obuchowski index of 0.9784. Detailed performance metrics included a macro-average F1-score of 0.89 (precision: 0.89, recall: 0.89) and a weighted-average F1-score of 0.89. Per-class performance, detailed in the confusion matrix, was also high (MS1: 99/116; MS2: 97/106; MS3: 116/130) (**Figure 5B**). To evaluate the reliability of the XGBoost multi-class classification model, we performed 100 independent random train/test splits. Multiple performance metrics confirmed its robustness: for example, the mean AUCs for MS1, MS2, and MS3 were 0.9756, 0.9746, and 0.9697, respectively. Detailed results are presented in **Figure 5C** and **Supplementary Table 6**.

### 3.5 Model interpretability and feature panel analysis

**Figure 6:**
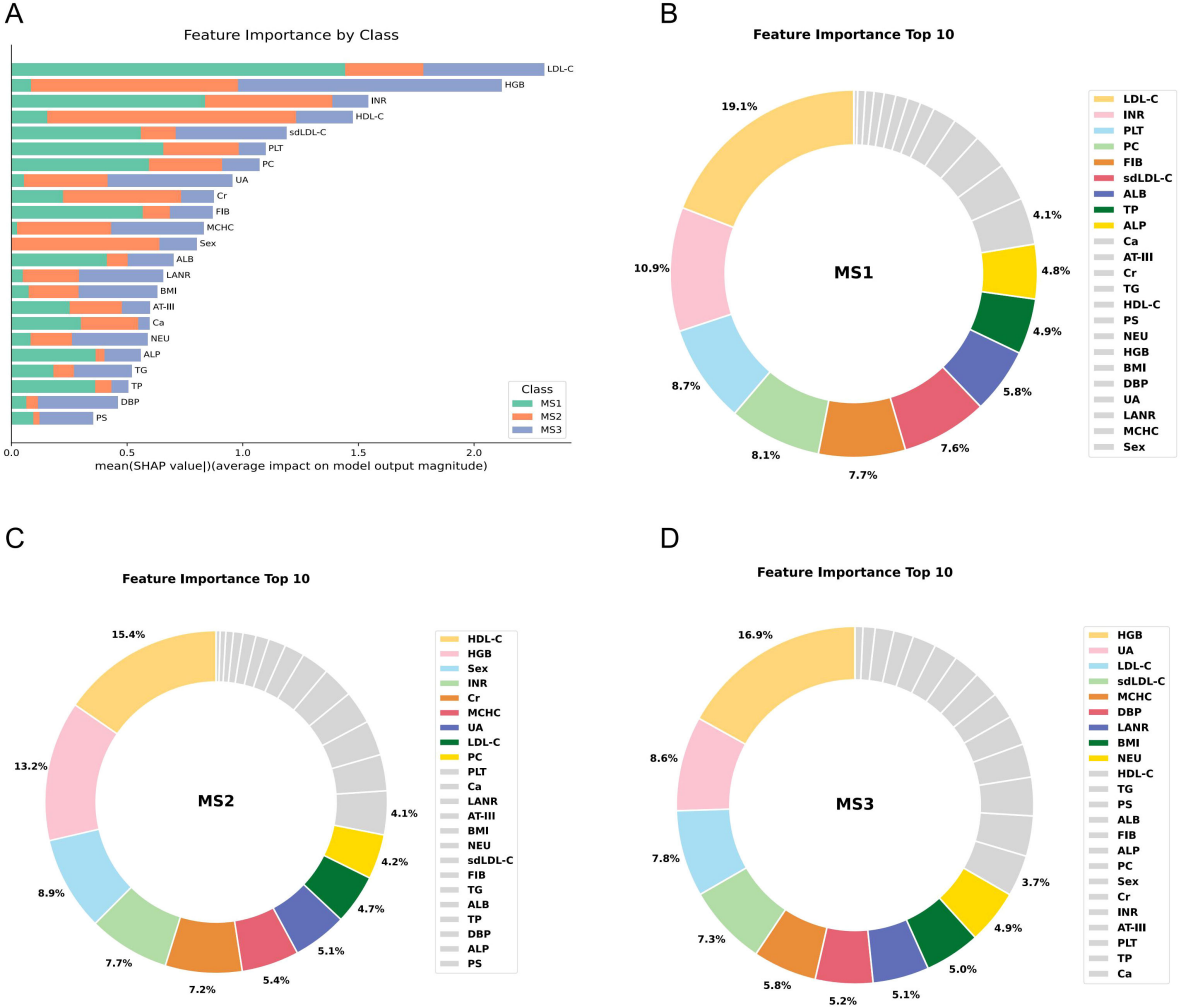
SHAP Interpretability analysis of subtype-defining features. **(A)** Feature Importance by Class. **(B–D)** Feature Contribution Donut Chart across three subtypes.

**Figure 7:**
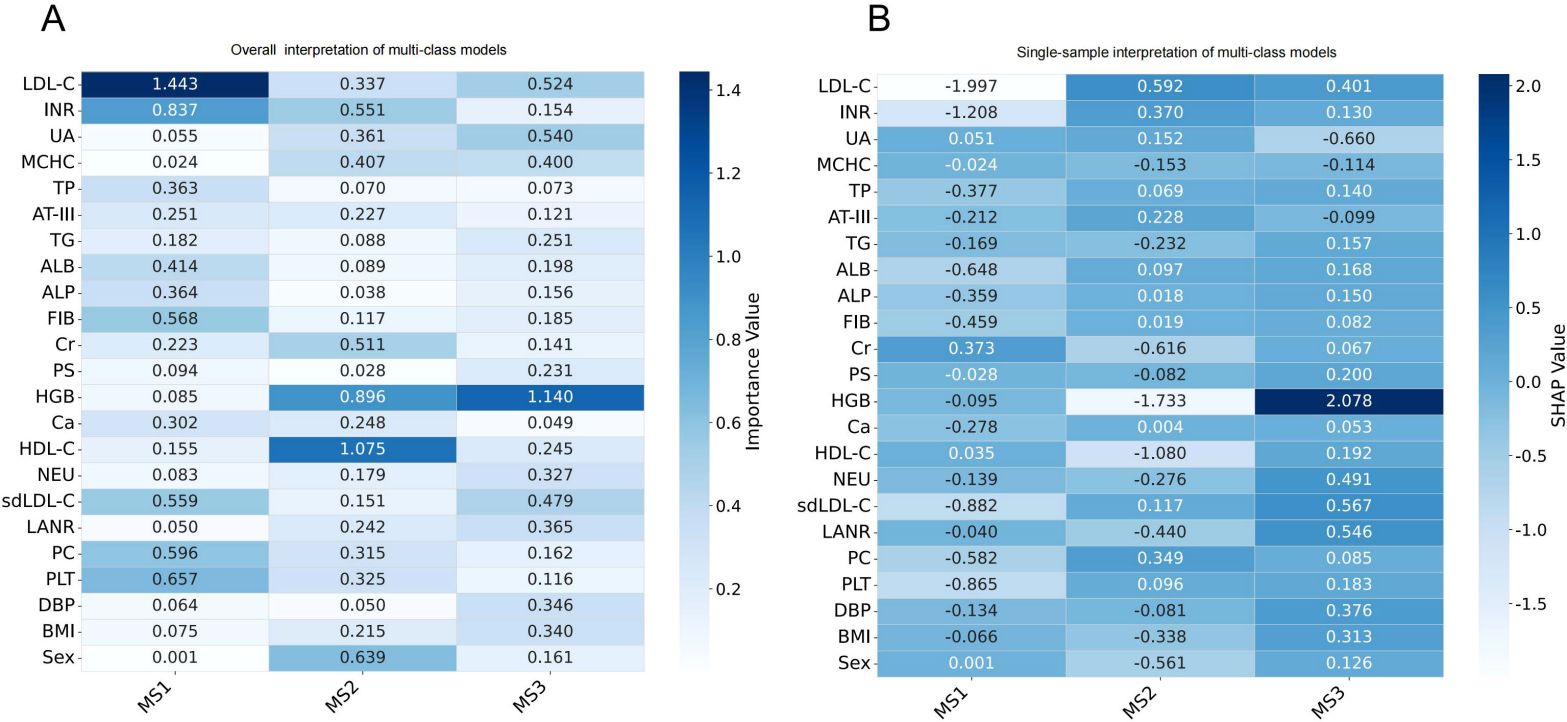
The SHAP heatmap shows the contribution of different features to the three-class classification model. **(A)** Feature importance distribution for each MS subtype. **(B)**This plot shows the SHAP values for an individual instance.

The SHAP analysis provided granular insights into the biological drivers of each subtype. Looking at the overall contribution of each feature, the top five most influential features were LDL-C, HGB, INR, HDL-C, and sdLDL-C(**Figure 6A**). Subtype-specific signatures emerged clearly: MS1 was primarily driven by elevated LDL-C, sdLDL-C, INR, FIB, PC, PLT and Ca (**Figure 6B**); MS2 was characterized by male predominance, higher Cr, UA, and HGB, but lower HDL-C and PLT (**Figure 6C**); MS3 was defined by female predominance, significantly lower BMI, UA, ALB, NEU, and HGB, but higher HDL-C (**Figure 6D**). Heatmaps and Beeswarm Plot illustrated the distinct prediction behaviors of these features across subtypes; while local SHAP explanations for individual samples demonstrated how specific patient values contributed to the model’s class assignment, facilitating case-by-case interpretability (**Figures 7A, B**, **Supplementary Figure 4**).

### 3.6 Webpage deployment tool

We implemented the XGBoost multi-class classification model into a web application (**Supplementary Figure 6**) that provides subtype predictions for MS patients based on the 23 selected blood and metabolic biomarkers. The application can calculate the probability of each MS subtype (MS1, MS2 and MS3) and exhibit the results on interactive visualizations to delineate explicit subtype classifications. The web application was made accessible online (https://ms-subtype.streamlit.app/), enabling clinicians to input patient laboratory data and receive immediate, objective stratification into one of the three pathophysiologically distinct MS subgroups identified in our study.

## 4 Discussion

We have defined three novel subtypes of MS, each with distinct hematological and metabolic profiles, through a data-driven biological framework. This objective approach, applied to a cohort of 1,756 patients using explainable machine learning, successfully shifts the subtyping paradigm away from reliance on subjective anatomical classification.

### 4.1 MS1 Subtype (hypercoagulable-inflammatory profile)

This subtype predominantly consisted of female patients (82.4%). The biomarker profile—characterized by elevated LDL-C, sdLDL-C, PLT, and FIB, with decreased INR and elevated PC—is consistent with a hyperlipidemic and hypercoagulable state. We hypothesize that this peripheral profile may be associated with cerebral small vessel dysfunction, though this inference requires direct validation through neuroimaging or histopathological studies.

Several lines of evidence support the biological plausibility of this hypothesis, while stopping short of establishing causation. First, oxidized LDL (oxLDL) has been shown to activate the lectin-like oxLDL receptor (LOX-1) on cerebral endothelial cells, triggering NF-κB-mediated upregulation of adhesion molecules, matrix metalloproteinase activation, and BBB disruption in animal models (17). Second, fibrinogen—significantly elevated in MS1—beyond its role in clot formation, is also a well-established microglial activation signal: extravasated fibrinogen binds Mac-1 (CD11b/CD18) integrin and TLR4 on microglia, inducing pro-inflammatory cytokine release and driving neuroinflammatory cascades (14). Third, platelets, traditionally viewed as hemostatic cells, are increasingly recognized as immune effector cells capable of promoting leukocyte extravasation and NETosis (25). The combined elevation of PLT and FIB suggests platelet-leukocyte interactions that may promote NETosis and cerebrovascular inflammation. Additionally, the protein C pathway, reflected by elevated PC in MS1, has well-characterized cytoprotective and anti-inflammatory functions via endothelial protein C receptor (EPCR)-mediated PAR1 signaling—its elevation may represent a compensatory anti-inflammatory response to the pro-coagulant, pro-inflammatory state (26).

Prior studies in cerebral small vessel disease have demonstrated associations between hypercoagulability, lipid abnormalities, and microcirculatory impairment in basal ganglia regions, particularly the globus pallidus and substantia nigra (27). However, we emphasize that the current data do not establish a causal chain from peripheral hypercoagulability to basal ganglia microcirculatory impairment in MS. Prospective studies incorporating neuroimaging markers of small vessel disease (e.g., white matter hyperintensities, cerebral microbleeds, diffusion tensor imaging) and cerebrospinal fluid (CSF) biomarkers of BBB integrity are needed to test this hypothesis.

### 4.2 MS2 subtype (metabolic-excess profile with impaired vascular protection)

This subtype showed a striking male predominance (76.1%), which we note may partially reflect physiological sex differences—higher Cr and HGB are expected in males due to greater muscle mass and higher androgen-driven erythropoiesis. Although the higher Cr in MS2 likely reflects normal sex-based differences, we cannot exclude the possibility that male sex may also represent a predisposing factor for certain MS subtypes—a question that warrants dedicated prospective investigation. Beyond these sex-related differences, the MS2 profile includes significantly lower HDL-C and distinct metabolic alterations that might suggest additional disease-associated mechanisms.

The low HDL-C levels may indicate impaired reverse cholesterol transport and reduced vascular anti-inflammatory protection. HDL particles normally suppress endothelial adhesion molecule expression, inhibit LDL oxidation, and modulate TLR signaling. Their reduction could therefore facilitate the pro-inflammatory effects of metabolic toxins on the cerebral vasculature. Previous studies have demonstrated that metabolic waste products, including uremic toxins and advanced glycation end-products, can directly compromise BBB integrity by disrupting endothelial tight junction proteins and promoting oxidative stress in the neurovascular unit (28, 29). We hypothesize that in MS2, the combination of metabolic excess (reflected by higher Cr and metabolic markers) and reduced vascular protection (low HDL-C) may create a state of heightened vulnerability to metabolic-toxin-induced BBB dysfunction. However, this hypothesis requires validation through direct measurement of BBB permeability markers and metabolic toxin levels.

The MS2 profile—male predominance, metabolic excess, low HDL-C—is consistent with the concept of “metaflammation,” a chronic low-grade inflammation driven by metabolic overload (30). The kidney-brain axis provides an additional mechanistic link, as the accumulation of metabolic toxins can directly compromise BBB integrity (28). We hypothesize that MS2 represents a “metabolic-immune” endophenotype, where metabolic excess and impaired vascular protection synergistically promote neurovascular vulnerability.

### 4.3 MS3 subtype (frailty-associated, antioxidant-deficient profile)

This subtype was predominantly female (93.8%) and characterized by significantly lower BMI, UA, ALB, HGB, and NEU. We acknowledge that this profile may partially reflect a frailty phenotype common in elderly female populations, rather than a MS-specific mechanism. However, several considerations suggest additional disease-relevant implications.

Uric acid is a major endogenous antioxidant, accounting for approximately 60% of plasma free radical scavenging capacity. At physiological concentrations, UA protects against peroxynitrite-mediated neuronal damage, preserves BBB integrity, and modulates neuroinflammation (18, 31). The significantly low UA levels in MS3 may therefore represent a state of depleted antioxidant reserve, consistent with our observation of concurrently decreased ALB (another major plasma antioxidant), TBIL, and GGT. Whether this antioxidant deficiency directly contributes to neuronal membrane instability and hypersensitivity in MS3 remains an open question requiring direct measurement of oxidative stress markers and neuronal excitability assessments. The low NEU in MS3 may indicate an immunosenescence/nutritional deficiency pattern. Albumin, as a negative acute-phase protein, reflects both chronic inflammatory status and nutritional adequacy. The combined low UA-ALB-NEU profile is consistent with reduced capacity for both antioxidant defense and immune surveillance, which could theoretically lower the threshold for neuronal hypersensitivity to external stimuli.

### 4.4 Clinical implications and future directions

The new data-driven classification of MS may inform future investigations into subtype-specific therapeutic strategies, though all such implications remain speculative pending prospective validation.

For MS1, the hypercoagulable-inflammatory profile suggests testable predictions: whether DBS, which delivers continuous electrical stimulation to the basal ganglia, shows differential efficacy in this subtype via potential effects on local microcirculation; and whether antiplatelet or lipid-lowering therapies provide symptomatic benefit—questions that can only be answered through subtype-stratified clinical trials. For MS2, the metabolic-excess profile suggests that lifestyle interventions targeting metabolic health might influence disease trajectory, though this has not been tested. For MS3, the antioxidant-deficient profile raises the question of whether nutritional supplementation and antioxidant strategies could improve neuronal stability.

Notably, we caution that these clinical implications are hypothesis-generating and should not be interpreted as treatment recommendations.

### 4.5 Limitations

This study has several important limitations. First, it’s a retrospective, single-center study from Henan Province, so the subtypes we identified may partly reflect local population characteristics rather than universal biology—multi-center validation is needed. Second, all our mechanistic interpretations are based solely on peripheral blood markers and remain inferential; they require confirmation from neuroimaging, CSF, or histology. Third, we didn’t systematically record medication use (antipsychotics, anticholinergics, benzodiazepines, botulinum toxin) or comorbidities (diabetes, hypertension, dyslipidemia), both of which could confound the biomarker profiles. Finally, the lack of CRP, cytokines, immune cell subsets, complement factors, and autoantibodies prevents us from directly demonstrating immune pathway involvement.

## 5 Conclusion

Three data-driven endophenotypes of MS were identified from comprehensive baseline data. Through integration with the neuroimmune literature, we propose testable hypotheses linking these peripheral profiles to potential central neuroimmune mechanisms, including fibrinogen-mediated microglial activation, oxLDL-driven BBB disruption, and uric acid-dependent antioxidant depletion. These findings provide a data-driven framework that may inform future prospective investigations into the precision medicine of MS, though substantial validation—including external multi-center replication, neuroimaging correlation, and direct immunological characterization—is required before clinical translation.

## Data Availability

The original contributions presented in the study are included in the article/**Supplementary Material**. Further inquiries can be directed to the corresponding author.
