# Supplementary material for: Use of explainable cluster analysis to identify distinct subtypes of Meige syndrome patients and associated biomarker profiles
Source: Front Immunol. 2026 Jul 30;17:1904682. doi: 10.3389/fimmu.2026.1904682 (PMC13469414; doi:10.3389/fimmu.2026.1904682)
Supplement: Supplementary file 1 [file DataSheet1.docx]

**Supplementary Materials: Use of Explainable Cluster Analysis to Identify Distinct Subtypes of Meige Syndrome Patients and Associated Biomarker Profiles**

**Contents**

[Supplementary Tables	2](#_Toc8974)

[Table S1. Detailed missing value statistics for all 66 initial variables	2](#_Toc5666)

[Table S2. Feature ranking based on statistical significance and effect size in MS1 VS Rest	3](#_Toc9707)

[Table S3. Feature ranking based on statistical significance and effect size in MS2 VS Rest	4](#_Toc18674)

[Table S4. Feature ranking based on statistical significance and effect size in MS3 VS Rest	4](#_Toc102)

[Table S5. The 23 key predictive features and their biological domains	5](#_Toc29022)

[Table S6. Performance Metrics of XGBoost over 100 Independent Random Splits	6](#_Toc11691)

[Supplementary Figures	7](#_Toc2965)

[Figure S1. Heatmap of missing data patterns before imputation	7](#_Toc14360)

[Figure S2. Kernel density estimation plots comparing the distribution of imputed variables before and after imputation.	8](#_Toc9150)

[Figure S3. Cluster Stability and Validation via 30 Bootstrap Resamples	9](#_Toc366)

[Figure S4. SHAP for the three-class XGBoost classification model.	10](#_Toc27112)

[Figure S5. LDL-C SHAP dependence plots	11](#_Toc16779)

[Figure S6. Convenient application for clinical utility.	12](#_Toc1626)

Supplementary Tables

Table S1. Detailed missing value statistics for all 66 initial variables

| **Characteristics** | **Missing proportion** | **Type** |
| --- | --- | --- |
| WBC | 14.14 | numerical |
| FT3 | 4.21 | numerical |
| PS | 3.7 | numerical |
| PC | 3.65 | numerical |
| LANR | 3.65 | numerical |
| AT-III | 3.65 | numerical |
| ApoA1 | 2.53 | numerical |
| TPOAb | 2.53 | numerical |
| TgAb | 2.53 | numerical |
| ApoB | 2.53 | numerical |
| sdLDL-C | 2.47 | numerical |
| HbA1c | 1.96 | numerical |
| FT4 | 1.96 | numerical |
| TSH | 1.96 | numerical |
| TG | 1.85 | numerical |
| LDL-C | 1.74 | numerical |
| TC | 1.74 | numerical |
| HDL-C | 1.74 | numerical |
| ALP | 1.46 | numerical |
| AST | 1.4 | numerical |
| ALT | 1.4 | numerical |
| TBA | 1.4 | numerical |
| GGT | 1.4 | numerical |
| TP | 1.4 | numerical |
| DBIL | 1.4 | numerical |
| ALB | 1.4 | numerical |
| TBIL | 1.4 | numerical |
| UA | 1.35 | numerical |
| BUN | 1.35 | numerical |
| Cr | 1.35 | numerical |
| FBG | 1.07 | numerical |
| Ca | 0.79 | numerical |
| K | 0.79 | numerical |
| Cl | 0.79 | numerical |
| Na | 0.79 | numerical |
| INR | 0.56 | numerical |
| TT | 0.56 | numerical |
| APTT | 0.56 | numerical |
| PT | 0.56 | numerical |
| FIB | 0.56 | numerical |
| MCHC | 0.28 | numerical |
| RDW-CV | 0.28 | numerical |
| RDW-SD | 0.28 | numerical |
| Lymphocyte | 0.28 | numerical |
| PDW | 0.28 | numerical |
| MPV | 0.28 | numerical |
| Eosinophilic | 0.28 | numerical |
| P-LCR | 0.28 | numerical |
| HCT | 0.28 | numerical |
| Basophils | 0.28 | numerical |
| Monocyte | 0.28 | numerical |
| Neutrophil | 0.28 | numerical |
| MCH | 0.28 | numerical |
| HGB | 0.28 | numerical |
| PLT | 0.28 | numerical |
| MCV | 0.28 | numerical |
| PCT | 0.28 | numerical |
| BMI | 0.06 | numerical |
| RBC | 0.06 | numerical |
| Pulse | 0 | numerical |
| DBP | 0 | numerical |
| SBP | 0 | numerical |
| Age | 0 | numerical |
| Sex | 0 | categorical |
| Hospital stay | 0 | numerical |
| Surgical history | 0 | categorical |

Table S2. Feature ranking based on statistical significance and effect size in MS1 VS Rest

| **Characteristics** | **Raw_pvalue** | **FDR_pvalue** | **Effect_size** | **Test** |
| --- | --- | --- | --- | --- |
| TC | 5.621E-169 | 3.5412E-167 | 1.57 | Student's t-test |
| ApoB | 7.1357E-161 | 2.2477E-159 | 1.53 | Student's t-test |
| LDL-C | 1.2362E-133 | 2.5959E-132 | 1.36 | Student's t-test |
| PC | 3.25863E-90 | 5.13234E-89 | 1.17 | Welch's t-test |
| sdLDL-C | 1.79479E-79 | 2.26143E-78 | 1.11 | Welch's t-test |
| TP | 8.16079E-64 | 8.56883E-63 | 0.94 | Welch's t-test |
| PLT | 2.02318E-47 | 1.82087E-46 | 0.76 | Student's t-test |
| INR | 1.15886E-46 | 9.126E-46 | 0.75 | Student's t-test |
| ALB | 1.93699E-46 | 1.35589E-45 | 0.75 | Student's t-test |
| PT | 2.62339E-46 | 1.65274E-45 | 0.75 | Student's t-test |
| PCT | 1.87341E-40 | 1.07295E-39 | 0.72 | Welch's t-test |
| Ca | 2.68299E-38 | 1.30022E-37 | 0.70 | Welch's t-test |
| AT-III | 2.19405E-38 | 1.15188E-37 | 0.70 | Welch's t-test |
| FIB | 1.3269E-32 | 5.97106E-32 | 0.62 | Student's t-test |
| ApoA1 | 4.20469E-26 | 1.76597E-25 | 0.54 | Student's t-test |
| PS | 7.61022E-24 | 2.99652E-23 | 0.52 | Student's t-test |
| ALP | 3.23031E-21 | 1.19711E-20 | 0.49 | Student's t-test |
| TG | 2.87744E-20 | 9.54099E-20 | 0.48 | Welch's t-test |
| TT | 2.86035E-20 | 9.54099E-20 | 0.47 | Student's t-test |
| Lymphocyte | 8.16415E-15 | 2.44925E-14 | 0.40 | Student's t-test |
| Cr | 4.4444E-15 | 1.39999E-14 | 0.37 | Welch's t-test |
| Cl | 1.93541E-12 | 5.5423E-12 | 0.36 | Student's t-test |
| DBP | 3.14501E-12 | 8.61459E-12 | 0.36 | Student's t-test |
| FBG | 1.11452E-08 | 2.60056E-08 | 0.34 | Welch's t-test |
| BMI | 8.96526E-11 | 2.35338E-10 | 0.33 | Student's t-test |
| Neutrophil | 7.59105E-09 | 1.83937E-08 | 0.31 | Welch's t-test |

Table S3. Feature ranking based on statistical significance and effect size in MS2 VS Rest

| **Characteristics** | **Raw_pvalue** | **FDR_pvalue** | **Effect_size** | **Test** |
| --- | --- | --- | --- | --- |
| HGB | 2.4466E-114 | 1.5413E-112 | 1.28 | Student's t-test |
| HCT | 7.59785E-98 | 2.39332E-96 | 1.17 | Student's t-test |
| Cr | 1.90075E-56 | 2.39494E-55 | 1.05 | Welch's t-test |
| HDL-C | 2.43997E-76 | 5.12394E-75 | 0.93 | Welch's t-test |
| ApoA1 | 1.59152E-71 | 2.50664E-70 | 0.92 | Welch's t-test |
| TC | 2.48593E-56 | 2.61022E-55 | 0.81 | Welch's t-test |
| UA | 4.08549E-44 | 3.21732E-43 | 0.79 | Welch's t-test |
| MCHC | 1.53808E-42 | 1.07665E-41 | 0.73 | Student's t-test |
| MCH | 1.03391E-46 | 9.30518E-46 | 0.66 | Welch's t-test |
| LANR | 6.97247E-27 | 3.13761E-26 | 0.65 | Welch's t-test |
| Sex | 5.9496E-158 | 1.7849E-157 | 0.64 | Pearson's Chi-square |
| Monocyte | 2.08731E-26 | 8.76671E-26 | 0.61 | Welch's t-test |
| INR | 7.63012E-25 | 2.82763E-24 | 0.60 | Welch's t-test |
| PT | 9.22516E-25 | 3.22881E-24 | 0.60 | Welch's t-test |
| AT-III | 2.31062E-29 | 1.32336E-28 | 0.60 | Student's t-test |
| DBIL | 1.63611E-20 | 4.90832E-20 | 0.60 | Welch's t-test |
| PCT | 3.50755E-31 | 2.20976E-30 | 0.59 | Welch's t-test |
| PLT | 6.07341E-29 | 3.18854E-28 | 0.57 | Welch's t-test |
| PC | 1.36046E-27 | 6.59302E-27 | 0.56 | Welch's t-test |
| ApoB | 5.10321E-25 | 2.00939E-24 | 0.51 | Welch's t-test |
| LDL-C | 5.23609E-23 | 1.64937E-22 | 0.50 | Welch's t-test |
| TBIL | 8.18208E-16 | 2.34305E-15 | 0.48 | Welch's t-test |
| MCV | 9.59747E-24 | 3.18232E-23 | 0.46 | Welch's t-test |
| BMI | 9.6094E-15 | 2.63214E-14 | 0.40 | Welch's t-test |
| DBP | 5.14858E-13 | 1.29744E-12 | 0.38 | Student's t-test |
| Neutrophil | 2.22788E-12 | 5.39832E-12 | 0.37 | Student's t-test |
| FIB | 2.32474E-12 | 5.42439E-12 | 0.37 | Student's t-test |
| TP | 2.85473E-13 | 7.49366E-13 | 0.37 | Welch's t-test |
| BUN | 3.59463E-12 | 8.08791E-12 | 0.36 | Student's t-test |
| Eosinophilic | 1.23587E-08 | 2.51162E-08 | 0.35 | Welch's t-test |
| RDW-SD | 2.11933E-10 | 4.4506E-10 | 0.33 | Student's t-test |
| Ca | 1.9239E-10 | 4.17951E-10 | 0.32 | Welch's t-test |

Table S4. Feature ranking based on statistical significance and effect size in MS3 VS Rest

| **Characteristics** | **Raw_pvalue** | **FDR_pvalue** | **Effect_size** | **Test** |
| --- | --- | --- | --- | --- |
| HGB | 4.7368E-128 | 2.9842E-126 | 1.26 | Welch's t-test |
| HCT | 4.5509E-117 | 1.4335E-115 | 1.19 | Welch's t-test |
| sdLDL-C | 4.49736E-87 | 9.44445E-86 | 0.95 | Welch's t-test |
| UA | 5.08245E-74 | 8.00486E-73 | 0.88 | Welch's t-test |
| LANR | 8.75344E-68 | 1.10293E-66 | 0.81 | Welch's t-test |
| ApoB | 4.01181E-61 | 4.2124E-60 | 0.78 | Welch's t-test |
| DBP | 3.39619E-44 | 2.6745E-43 | 0.71 | Student's t-test |
| BMI | 2.44446E-43 | 1.54001E-42 | 0.70 | Student's t-test |
| LDL-C | 1.12839E-47 | 1.01555E-46 | 0.69 | Welch's t-test |
| PS | 1.56967E-43 | 1.09877E-42 | 0.66 | Welch's t-test |
| Neutrophil | 5.73605E-41 | 3.01142E-40 | 0.65 | Welch's t-test |
| MCHC | 1.39432E-31 | 6.27444E-31 | 0.62 | Welch's t-test |
| TG | 9.64574E-43 | 5.52438E-42 | 0.60 | Welch's t-test |
| HDL-C | 1.66119E-30 | 6.15618E-30 | 0.60 | Welch's t-test |
| Monocyte | 1.25288E-35 | 6.07164E-35 | 0.59 | Welch's t-test |
| TC | 7.48277E-31 | 2.94634E-30 | 0.54 | Welch's t-test |
| ALB | 1.22755E-25 | 3.51526E-25 | 0.53 | Student's t-test |
| Cr | 3.60069E-31 | 1.51229E-30 | 0.53 | Welch's t-test |
| TP | 8.36464E-26 | 2.63486E-25 | 0.51 | Welch's t-test |
| PC | 2.27457E-26 | 7.54199E-26 | 0.50 | Welch's t-test |
| MCH | 2.54437E-19 | 6.67897E-19 | 0.50 | Welch's t-test |
| TT | 5.04374E-23 | 1.38155E-22 | 0.50 | Student's t-test |
| FBG | 1.52968E-27 | 5.35389E-27 | 0.47 | Welch's t-test |
| ALT | 1.17733E-25 | 3.51526E-25 | 0.46 | Welch's t-test |
| Lymphocyte | 7.83837E-19 | 1.97527E-18 | 0.43 | Welch's t-test |
| Sex | 1.23487E-65 | 3.7046E-65 | 0.41 | Pearson's Chi-square |
| TBIL | 1.00766E-17 | 2.3512E-17 | 0.40 | Welch's t-test |
| GGT | 3.28835E-18 | 7.96793E-18 | 0.37 | Welch's t-test |
| Cl | 2.60206E-14 | 5.85463E-14 | 0.37 | Welch's t-test |
| Ca | 7.22615E-13 | 1.56982E-12 | 0.35 | Welch's t-test |
| MCV | 1.07132E-09 | 2.04526E-09 | 0.33 | Welch's t-test |
| SBP | 1.65495E-10 | 3.4754E-10 | 0.32 | Student's t-test |

Table S5. The 23 key predictive features and their biological domains

|  | **Feature** | **Biological Domain** |
| --- | --- | --- |
| 1 | Low-density lipoprotein-cholesterol (LDL-C) | Lipid metabolism |
| 2 | High-density lipoprotein-cholesterol (HDL-C) | Lipid metabolism |
| 3 | Small-dense low-density lipoprotein cholesterol (sdLDL-C) | Lipid metabolism |
| 4 | Triglycerides (TG) | Lipid metabolism |
| 5 | International normalized ratio (INR) | Coagulation |
| 6 | Fibrinogen (FIB) | Coagulation |
| 7 | Antithrombin III (AT-III) | Coagulation |
| 8 | Protein S (PS) | Coagulation |
| 9 | Platelet Count (PLT) | Coagulation/Immune |
| 10 | Protein C (PC) | Coagulation |
| 11 | Lupus anticoagulant normalization rate (LANR) | Coagulation/Immune |
| 12 | Uric Acid (UA) | Renal/Metabolic |
| 13 | Serum creatinine (Cr) | Renal/Metabolic |
| 14 | Albumin (ALB) | Nutritional/Immune |
| 15 | Total protein (TP) | Nutritional |
| 16 | Serum calcium (Ca) | Metabolic |
| 17 | Alkaline phosphatase (ALP) | Metabolic |
| 18 | Hemoglobin (HGB) | Hematology |
| 19 | Mean Corpuscular Hemoglobin Concentration (MCHC) | Hematology |
| 20 | Neutrophil (NEU) | Hematology/Immune |
| 21 | Sex | Demographic |
| 22 | Body mass index (BMI) | Anthropometric |
| 23 | Diastolic blood pressure (DBP) | Cardiovascular |

Table S6. Performance Metrics of XGBoost over 100 Independent Random Splits

| **Metric** | **Mean** | **Std Dev** | **95% CI** |
| --- | --- | --- | --- |
| MS1-AUC | 0.9756 | 0.0063 | [0.9743,0.9768] |
| MS2-AUC | 0.9746 | 0.0062 | [0.9734,0.9759] |
| MS3-AUC | 0.9697 | 0.007 | [0.9683,0.9711] |
| Macro-Average-AUC | 0.9743 | 0.005 | [0.9733,0.9752] |
| ObuchowskiIndex | 0.9733 | 0.0051 | [0.9723,0.9743] |
| Accuracy | 0.8629 | 0.0182 | [0.8593,0.8665] |
| Macro-Precision | 0.8657 | 0.018 | [0.8621,0.8693] |
| Macro-Recall | 0.8609 | 0.0184 | [0.8572,0.8645] |
| Macro-F1 | 0.8623 | 0.0183 | [0.8587,0.8660] |
| Kappa | 0.7933 | 0.0276 | [0.7878,0.7987] |
| LogLoss | 0.3661 | 0.0205 | [0.3620,0.3701] |

Supplementary Figures

Figure S1. Heatmap of missing data patterns before imputation

Figure S2. Kernel density estimation plots comparing the distribution of imputed variables before and after imputation.

Figure S3. Cluster Stability and Validation via 30 Bootstrap Resamples

Figure S4. SHAP for the three-class XGBoost classification model.

Beeswarm plot illustrates the global feature importance ranking for predicting different subtypes; waterfall plot demonstrates the contribution of each feature to the predicted class of individual samples.

Figure S5. LDL-C SHAP dependence plots

Figure S6. Convenient application for clinical utility.

(A convenient XGBoost three-class classification model with 23 features is available for MS subtype prediction. Upon entering the actual values of the 23 features, the application automatically displays the predicted probabilities and clinical interpretation.)
